# Platelet Defects in Acute Myeloid Leukemia—Potential for Hemorrhagic Events

**DOI:** 10.3390/jcm11010118

**Published:** 2021-12-26

**Authors:** Horia Bumbea, Ana Maria Vladareanu, Ion Dumitru, Viola Maria Popov, Cristina Ciufu, Anca Nicolescu, Minodora Onisai, Cristina Marinescu, Diana Cisleanu, Irina Voican, Sinziana Sarghi

**Affiliations:** 1 Department of Hematology, Emergency University Hospital, 050098 Bucharest, Romania; horiabum@gmail.com (H.B.); anamariavladareanu@yahoo.com (A.M.V.); punctdoc@yahoo.com (I.D.); cciufu@yahoo.com (C.C.); apbv24@yahoo.co.uk (A.N.); minodorel@yahoo.com (M.O.); mecrystyna13@yahoo.com (C.M.); dcisleanu@yahoo.com (D.C.); voicanirina@yahoo.com (I.V.); 2 Department of Hematology, Carol Davila University of Medicine and Pharmacy, 020021 Bucharest, Romania; 3 Department of Hematology, Colentina Clinical Hospital, 020125 Bucharest, Romania; 4 (VP) Centre, Hospitalier René Dubos, 6 Avenue de l’île de France, 95300 Pontoise, France; ssarghi@yahoo.com

**Keywords:** platelet adhesion, platelet activation, acute myeloid leukaemia, flow cytometry

## Abstract

Background and objectives: In acute myeloid leukemia (AML), extensive bleeding is one of the most frequent causes of death. Impaired activation and aggregation processes were identified in previous studies on platelet behaviour associated with this disease. This study’s aim was to examine platelet function in correlation with other haemorrhage risk factors (fever, sepsis, recent bleeding, uraemia, leucocytosis, haematocrit value, treatment). Design and methods: The analysis of platelet surface proteins (Glycoprotein Ib-IX (CD42b, CD42a), Glycoprotein IIb-IIIa (CD41, CD61), *p*-selectin (CD62P), granulophysin (CD63)) was conducted by flowcytometry from samples of whole blood in patients with acute myeloid leukaemia in different stages of diagnosis and therapy (*n* = 22) in comparison with healthy human controls (*n* = 10). Results and interpretations: Our results show a significant decrease in fluorescence level associated with platelet activation markers (CD63 (14.11% vs. 40.78 % *p* < 0.05); CD62P (15.26% vs. 28.23% *p* < 0.05)); adhesion markers (CD42b (69.08% vs. 84.41% *p* < 0.05)) and aggregation markers (CD61 (83.79% vs. 98.62% *p* < 0.001)) in patients compared to controls. The levels of CD41 (80.62% vs. 86.31%, *p* = 0.290) and CD42a (77.98% vs. 94.15%, *p* = 0.99) demonstrate no significant differences in the two groups. Conclusion: The AML patients present changes in adhesion receptors and activation markers, suggesting a functional defect or denatured intracellular signalling in platelets. The exposed data indicate that flow cytometry can effectively identify multiple functional platelet impairments in AML pathogenesis.

## 1. Introduction

Acute myeloid leukaemia (AML) is a clonal haematopoietic stem cell disease [1,2] characterized by bone marrow infiltration with leukemic myeloid blast cells and low counts of other hematopoietic lineages, producing leucopenia, anemia and thrombocytopenia. AML is frequently associated with a life-threatening haemorrhage. Haemorrhagic complications are a very frequent part of the clinical picture of acute myeloid leukemia. The main causes of this complication are thrombocytopenia and/or defective platelet function, abnormalities of coagulation or fibrinolysis process. It was proven that the high expression of activated GPIIbIIIa, especially p selectin and CD 63, are present in AML patients with a bleeding history. Abnormalities of platelet aggregation were also associated with haemorrhagic complications in the past. Thrombocytopenia is not correlated with a high incidence of bleeding in AML patients [3]. Abnormalities of coagulation and fibrinolysis are present especially in APL but also, in rare cases, in other subtypes of AML such as AML [4,5]. The frequency of disseminated coagulation (DIC) in hematologic malignancies is not low (12.7%), and one half of these patients had an unfavourable evolution, haemorrhage being the main cause of mortality in these patients [4]. Fibrinolysis is one of the most important factors involved in haemorrhagic complication in AML. Both mechanisms, secondary and primary, of DIC, such as high levels of urokinase-type plasminogen activator (u-PA), annexin-II, tissue-type plasminogen activator (tPA), reduced the levels of plasminogen and α2-antiplasmin, and were present in APL (acute promyelocytic leukemia). Annexin–II, the receptor for plasminogen and tPA, is highly expressed in endothelial cells of cerebral microvasculature, and, likely due to this expression, the incidence of intracerebral haemorrhage is more frequent. Elastase and chymotrypsin from myeloid blasts are involved in the proteolysis of proteins of the coagulation cascade (clotting factors and fibrinogen). Cytokines delivered by leukemic promyelocytes induce APL coagulopathy. These are represented by IL-1β and TNFα and secondary the loss of the thrombomodulin–anticoagulant cofactor [5].

Mucosal bleeding, petechiae, ecchymosis, fundal or cerebral hemorrhages may occur as a result of thrombocytopenia or thrombocytopathia. Previous research on platelet role in bleeding phenomenona of acute leukaemia patients identified defects in platelet production [6,7] and impaired platelet function [8,9,10,11,12]. These studies were particularly focused on aggregation defects [13,14,15] and bleeding at diagnosis [16]. Even if the risk of bleeding was correlated with platelet count in AML, it was shown that platelet aggregation and activation tests were more important in the evaluation of haemorrhagic complication than platelet count alone [3]. On the other hand, aggregation studies on platelets are very difficult to perform in severe thrombocytopenia, where this method has technical limitations [17].

From analysing the literature, we found that platelet function analysis using whole-blood flow cytometry was performed in a relative small number of studies [18,19,20,21] and in a rigorously selected group of patients [19,22].

The occurrence of haemorrhages in AML patients was thought to be due to additional risk factors, including fever, sepsis [20,23], anticoagulant therapy and drugs [20,24,25], coagulation anomalies, hypoalbuminemia [26], uraemia [27,28], recent bone marrow transplants, low haematocrit [29,30], leukopenia or leucocytosis [20], and vascular integrity [20]. Data regarding the independent predictive role of these factors in acute leukaemia patients, and for the estimation of severity of the bleeding, are very poor.

Consequently, the goal of our research is to extend the flow cytometry examination of platelet activation (CD63, CD62p,) adhesion (CD42a, CD42b) and aggregation (CD41, CD61) markers to the particular clinical context of the patient with AML.

## 2. Materials and Methods

The analysed group was composed of 22 patients diagnosed with AML at the Bucharest Emergency University Hospital and Colentina Clinical Hospital; all patients undergoing treatment specific for the stage of the disease. The patients’ diagnosis was established by bone marrow examination in accordance with World Health Organization classification [31], and the treatment was conducted according to international protocols. The AML classification was conducted using French-American-British (FAB) system [32,33] because cytogenetic and molecular analysis was not available during the period in which the patients were enrolled in the study, and usually the therapeutic approach was conducted in relation to FAB classification during the study period. Ten healthy volunteers recruited from the hospital staff members were enrolled as controls.

### 2.1. Monoclonal Antibodies

The following six monoclonal antibodies were used for the identification of platelet defects: CD61 clone SZ21 IM1758 (FITC conjugated) [34], CD41 clone P2IM1416 (PE conjugated) [33], CD42a clone SZ1IM1757 (FITC conjugated), CD42b clone SZ2IM1417 (PE conjugated), CD62P clone CLBThromb/6IM1164 (FITC conjugated) and CD63 clone CLBGran/12IM1914 (PE conjugated)—all were produced by Coulter-Immunotech [31].

Anti CD61 antibody is directed against the Glycoprotein [Gp] IIIa (β3), sub-unit of the Gp IIb-IIIa, the most frequent platelet surface integrin. The CD41 antigen (αIIb chain from integrin) is always non-covalently associated with the CD61. CD41 is expressed by platelets, megakaryocytes and a small subset of CD34^+^ cells, suggesting that CD41/CD61 is one of the earliest markers of the megakaryocytic lineage. The P2 antibody reacts with CD41a (GpIIbα) in the intact complex with GpIIIa but not with the GpIIb or GpIIIa separately.

Anti CD42b antibody is targeted against Gp Ib-α chain, the moiety of the von Willebrand receptor, GpIb. GpIbα, GpIbβ, GpIX (CD42a), and GpV represent a multifunctional receptor (the glycoprotein Ib-V-IX). The CD42a/CD42b complex is the receptor for von Willebrand’s factor and is known as von Willebrand factor-dependent adhesion receptor. CD42a expression is restricted to platelets and megakaryocytes. The SZ1 monoclonal antibody reacts with the complex CD42a/CD42b but does not recognize GpIb or GpIX individually [35].

Anti CD 62P antibody is specific for p selectin, a protein present in megakaryocytes, in Weibel–Palade bodies of endothelial cells and in alpha granules of platelets. It is translocated to the platelet surface membrane upon platelet activation [36]. The p-selectin role in vivo is still under study but there is strong evidence that it interferes in leukocyte recruitment and potentially supports platelet rolling [37,38,39].

Anti CD63 antibody is directed against Gp53 (granulophysin), a four transmembrane protein member of the tetraspanin (TM4SF) family present on the membranes of dense granules and lysosomes b. It relocates to the plasma membrane following platelet activation and degranulation. On the membrane surface it associates with the platelet integrin αIIbβ3-CD9 complex and with the actin cytoskeleton [40].

### 2.2. Preparation of Whole Blood Samples for Flow Cytometry

Blood samples (3 mL peripheral blood) from patients (*n* = 22) and volunteers (*n* = 10) were used for analyses. The samples were collected by venous punction from the antecubital vein with a minimum of stasis in Beckton Dickinson vacutainers with anticoagulant sodium citrate. The analysis was performed within 4–6 h from the venous punction with a FACS Calibur BD four channels flowcytometer, using the CellQuest Software.

The protocol for the platelet immunophenotyping is described below:

1. The blood samples were centrifuged at 20 °C and 200× *g* for 10 min, and the platelet-rich plasma obtained was removed and centrifuged at 20 °C and 800× *g* for 10 min to prepare the platelet pellet. The platelet pellet was washed three times with phosphate-buffered saline (PBS) containing EDTA (0.009 mol/L Na2 EDTA; 0.01 mol/L Na2 HPO4; 0.0018 mol/L KH2PO4; 0.17 mol/L NaCl and 0.0033 mol/L KCl), and then fixed by incubation for 10 min at room temperature with 2% paraformaldehyde in PBS-EDTA. The fixed platelets were washed twice with PBS-EDTA and adjusted to a concentration of 5 × 109/L. Aliquots (200 µL) of the platelet suspension were added to 12 mm × 75 mm polystyrene tubes previously coated with 30 µL of 5% bovine albumin.
2. The platelets were incubated for 15 min with the above-described monoclonal antibodies conjugated with fluorocromes for surface receptors

### 2.3. Flow Cytometry Analysis of Platelets in Whole Blood

The flow cytometry settings were optimized for the acquisition of platelets by logarithmic signal amplification in all detectors. Electronic compensation was used to control for spectral overlap and the flow cytometer was aligned daily with CaliBriteTM beads (BDIS). A threshold on FL-1 was set to ensure that only platelet CD61-positive events were collected. A minimum of 5000 CD61-positive platelets was collected from each sample.

**Figure 1:**
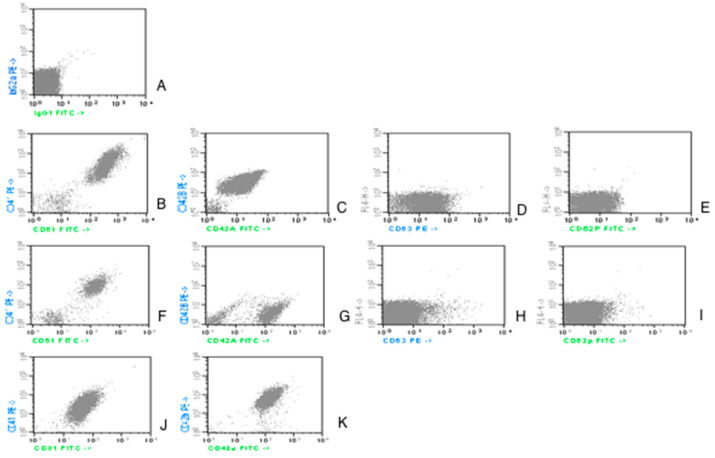
Dot-plot acquisition histograms representing expression of CD61 and CD41 on platelets in AML patients study group.

For analysis, an electronic gate enclosing platelets was set, as defined by forward scatter (FSC) and 90° side scatter (SSC) characteristics and CD61/CD41 platelet positivity markers, excluding platelet-leukocytes aggregates as well as platelet-erythrocyte and platelet-leukocytes doublets [18]. Further analysis of platelet activation markers CD62P, CD63, CD42b, and CD42a was performed on non-aggregated platelets as defined in Figure 1. Cells stained with an isotype control antibody were used as a reference when defining the threshold in order to distinguish negative from positive cells.

The platelet fluorescence of activation markers was measured as percentage of platelets expressing fluorescence above a threshold set to include only 1% of platelets incubated with the respective control.

Counting from the upper part of the picture and from the left side:

- the first line, image A, is an isotype control;
- the second line, a case of control group; image B for CD61/CD41; C for CD42a/CD42b; D for CD63; E for CD62P;
- the third and fourth lines represent AML patients. The images are illustrative for the variability in expression:

Images F and G from line 3 illustrate the expression of the aggregation (CD41/CD61) and adhesion (CD42a/CD42b) markers. In these examples, CD61, CD41 and CD42b have a lower fluorescence level in patients vs. controls.

Images J and K from line 4 show a different expression pattern with CD42b expression, which is as high as the expression at the controls.

Images H and I from line 3 present the fluorescence level for the activation markers (CD63, CD62P): reduced in patients vs. controls.

The acquisition was made with BD FACS Calibur flowcytometer and the analysis performed with Cellquest Software, from Becton Dickinson and Company, 1997, Becton Dickinson Immunocytometry Systems, San Jose, CA 95131, United States of America. Reagents were produced by IMMUNOTECH SAS, a Beckman Coulter Company, Marseille, France.

### 2.4. Statistical Analysis

For the medians, the 25th and 75th quartiles of the data were used to describe the observed values. Antigen expressions were considerate, continuous variables and were compared using non-parametric Wilcoxon rank sum test.

Spearman’s rank correlation was used for correlation analyses between platelets’ surface antigens and values of haematocrit, leukocytes, platelets, the percentage of peripheral blasts, level of uric acid, creatinine, BUN, and body temperature, all considered continuous variables. A general two-tailed significance level of 5% was applied.

Other clinical determinants, such as the presence of infection (localized or generalized) or cutaneous haemorrhagic syndrome, were analysed using crosstabs procedure.

All analyses were performed using SPSS statistical software, version 12.0 for Windows.

## 3. Results

The present study examines a group of 22 patients (13 females and 9 males) with ages ranging between 28 and 83 years. At the time of the analysis, they were already diagnosed with acute myeloid leukaemia and in different stages of disease.

Each patient was investigated by clinical examination, full blood count and peripheral blood smear with a detailed lymphocyte count. In every case, biochemistry samples (uric acid, creatinine, BUN) were taken and analysed.

As the majority of the patients were hospitalised at the time of the study, information about the treatment received a day before collecting the probes was available: chemotherapy, antibiotic therapy (beta-lactam antibiotics) and blood products transfusion therapy (Table 1).

The diagnosis of acute myeloid leukaemia was established prior to the beginning of the study, and the patients were assessed against AML subtypes according to FAB classification and EGIL criteria for AML0 [33,41]. Almost half of the group had AML type 4 (40.9%) and one-third (27.27%) had AML type 2. The AML type 3 FAB (promyelocytic) patients were excluded from the study design, due to frequently associated coagulation disorders. The number of cases with AML type 1 and AML type 0 were comparable (18.18% versus 13.64%).

The presence or absence of infection (localized or generalized), body temperature and cutaneous haemorrhagic syndrome were clinically assessed. Fever was present in almost half of patients with infection. Skin haemorrhage was present in 59% of AML patients with no significant difference according to AML subtype.

**Figure 2:**
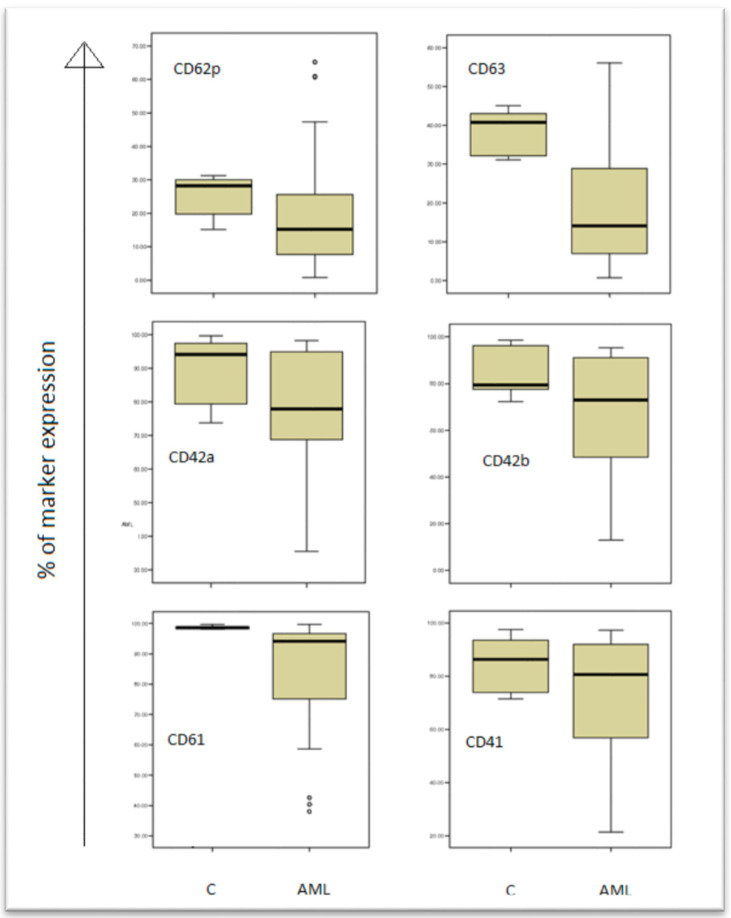
Expression of platelet activation markers in acute myeloid leukaemia (AML) patients and controls (C).

Platelets’ antigen expressions (percentage fluorescence above a threshold set to include only 1% of platelets incubated with control) had an abnormal, non-homogenous distribution in patient group (skewness and kurtosis almost equal to ±1). The medians and the 25th–75th percentiles were used to appreciate and compare distributions in patients and human control groups (Table 2, Figure 2).

The results are presented in box plots indicating the median as the horizontal line, 25th–75th percentiles in the group distributions as boxes, and 2.5–97.5% cumulative frequencies as whiskers. Outliners (identified by the 1.5 × inter-quartile range (IQR) criterion) are plotted as empty squares: CD62P = *p*-selectin; CD63 = granulophysin.

The control arm (C), and patients arm (AML) are shown in the boxplots, and the expression of all markers (CD61, CD41, CD42a, CD42b, CD62p, CD63) are decreased in AML patients comparing to controls.

### 3.1. Activation of Platelets in AML

**Figure 3:**
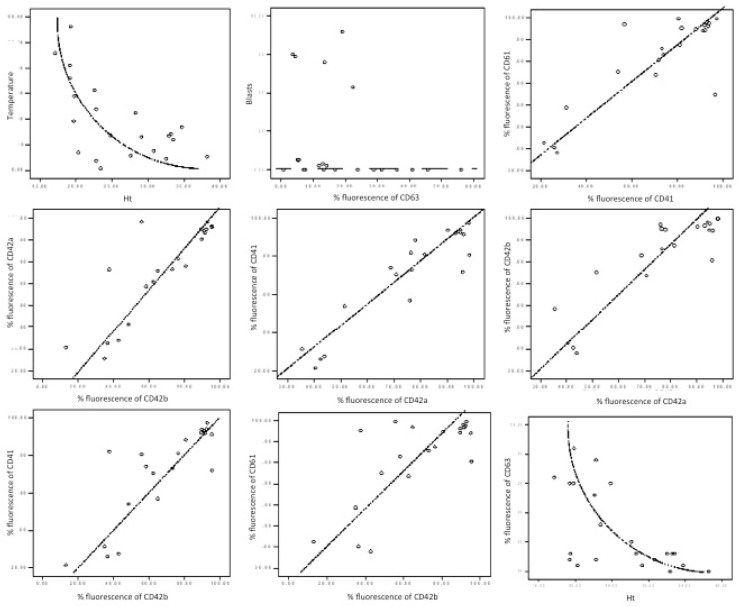
Graphical correlation in AML patients. Each box illustrates the statistic results presented in Table 3. There are represented correlations between temperature and haematocrit (Ht)—non-linear inverted correlation; CD63 and blasts—no correlation; CD41 and CD61—direct correlation; CD42a and CD42b—direct correlation; CD42a and CD41—direct correlation; CD42b and CD42a—direct correlation; CD42b and CD41—direct correlation; CD42b and CD61—direct correlation; Ht and CD61—inverted correlation.

The *p*-selectin level was found to be significantly reduced (15.26% versus 28.23%, *p* = 0.038) in patients versus controls. The CD63 (granulophysin) level correlates with the CD62P (*p* selectin) level (Table 3 and Figure 3: Rho = 0.69, *p* value < 0.01), and it had a decreased level in AML patients versus controls (14.11% vs. 40.78%).

A non-linear negative correlation was found between CD63 fluorescence percentage and haematocrit level (Table 3, Figure 3: Rho = −0.598, *p* value = 0.003).

**Figure 4:**
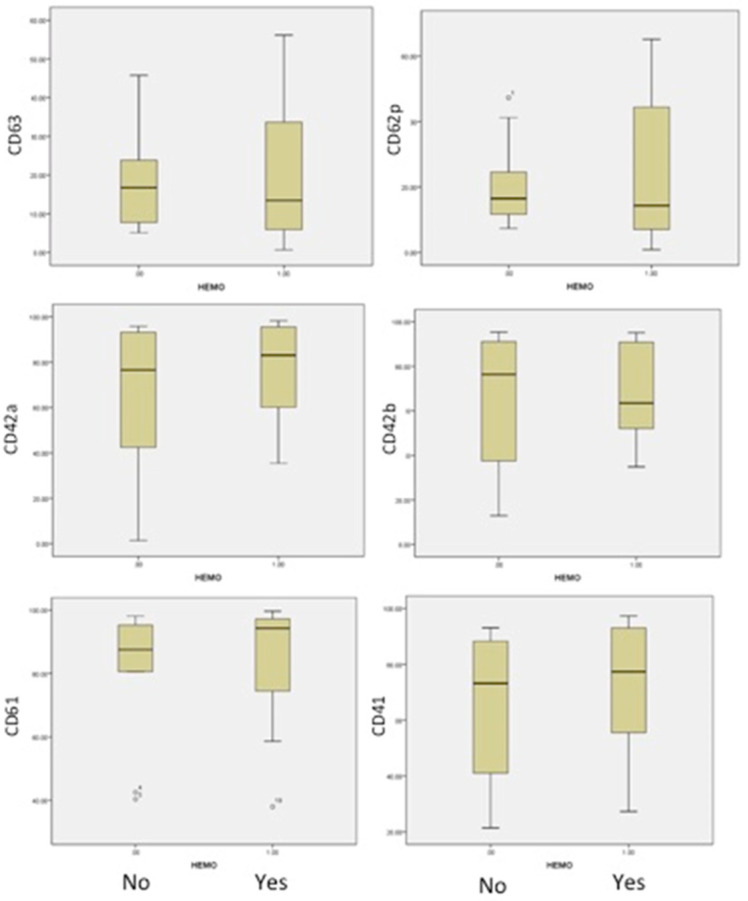
The flow cytometric analysis of our 22 AML patients and 10 controls revealed the following platelet characteristics. Patients without bleeding (arm No) or with bleedings (arm Yes) are represented in the boxplots with the level of expression for platelets markers CD61, CD41, CD42a, CD42b, CD62p and CD63.

The analysis of AML patients, with or without bleeding, was conducted in correlation with the number of platelets and their antigen expressions. The results are presented in Table 4. The medians and 25th–75th percentiles were used to appreciate and compare distributions in both groups (Figure 4). The median age was closely similar in both groups: 46 and 43 years old.

### 3.2. Von Willebrand Receptor in AML

The CD42b expression decreases in patients versus controls (*p* = 0.047), while the anti-CD42a antigen antibody, which reacts with the whole CD42a/CD42b complex, presents no particular difference in the study group versus the control group.

### 3.3. Fibrinogen Receptor

Gp IIb-IIIa, β3 integrin (CD61), the most frequent platelet surface antigen, presents a significant decrease in fluorescence percentage levels (94.08% vs. 98.67%, *p* value < 0.01) in patients versus controls (Table 3).

An interesting relation between Gp IIb-IIIa and Gp Ib-IX occurred (Table 3). Even if the connection between the two protein complexes had a variable strength (Rho: 0.570–0.783), the *p* value < 0.01 indicated that, in patients with AML, the expression of these two platelet determinants was related, likely as a global defect of signal transduction.

### 3.4. Comparison between Patients with Bleeding and without Bleeding

An important difference was identified in patients with bleeding, in comparison to patients without bleeding, in the form of a decrease in Gp Ib-IX expression (CD42a/CD42b) and the activation of less platelets (CD62P and CD63), as shown in Figure 4. This suggested a more severe acquired Bernard–Soulier syndrome and the blocking of platelet activation, independent of patient age or the number of platelets. The expression of fibrinogen receptor (CD61/CD41) was found with a higher level of expression, even if a lower level of expression in this receptor was found in all AML patients compared to healthy human volunteers. Our findings are strong evidence that haemorrhagic events in acute myeloid leukemias are more related to the impaired function of platelets.

### 3.5. Clinical and Laboratory Characteristics

Proven sepsis represents a significant statistic predictor of haemorrhage and correlates with the increased clinically significant bleeding risk the following day, as well as with the refractoriness risk to platelet concentrate transfusion. Our data revealed a statistically significant correlation between the haematocrit level and body temperature (ro: 0.559, *p* value: 0.05). A cutaneous haemorrhage was non-statistically correlated with the number of platelets. No correlation could be established between coagulation factors or uraemia markers and the other determinants. In the control group, no significant correlation was found between the studied variables.

We could not analyse the correlations between the different expression of platelets markers and AML subgroups because of the small number of patients in each subgroup.

## 4. Discussion

The bleeding predisposition in AML patients is the result of a platelet production defect leading to thrombocytopenia and the qualitative changes of the megakaryocytes and platelets, respectively [38,39]. Recent studies reported the more significant role of platelet dysfunction than the number of platelets for predicting haemorrhagic complications in AML patients [3]. Impaired platelet aggregation tests and lower expressions of GPIIb-IIIa and *p* selectin (CD62P) in platelets stimulated by TRAP (thrombin-receptor stimulating peptide) represent predictive factors for bleedings in AML [3].

Early studies that concentrated on the relationship between haemorrhage and thrombocytopenia concluded that bleeding was considered a frequent complication of induction chemotherapy or stem-cell transplants in acute myeloid leukaemia.

The most frequently observed platelet dysfunctions associated with haematological diseases are: inadequate platelet aggregation to ADP, epinephrine or collagen; low nucleotides secretion; low serotonin secretion; a decrease in thromboxane production and low PDGF and beta-thromboglobuline production [42,43,44,45]; the overproduction of cAMP [46]; and platelet signalling defects.

The reduced level of CD63 and CD62P in our patients indicated two possible events: (1) no activation process took place or (2) there was a brief activation followed by rapid *p* selectin secretion.

In addition to the above-mentioned studies, which were focused on functional platelets’ characteristics, our research performed on additional patient platelets that were not further stimulated, revealed the same low level of activation markers mentioned before. These results raise the hypothesis of the lack of platelets activation in vivo, a comparable state with the one found in vitro by the other experiments. Furthermore, the activation of platelets represents a vital first step in the clotting cascade.

The haematocrit role in platelets activation was previously investigated using in vitro stimulations [47]. Eugster M and Reinhart W found an inverse correlation between haematocrit and a necessary amount of time for the formation of the platelet plug. Our results show an inversely proportional relation between the haematocrit and granulophysin expression on platelets, which may be a false statistical result with no biological signification or a future research perspective.

Specific heterozygous mutations affecting the hematopoietic transcription factor CBFA2 were previously reported to be associated with a familial platelet disorder predisposing to acute myeloid leukaemia [48]. Our study correlated the percentage of the peripheral blast cells with platelet-specific markers. Although peripheral blasts seemed statistically related to CD63, this conclusion could not be graphically validated (Figure 3). This fact imposes a larger number of cases and, potentially, a different analytic strategy.

### 4.1. Von Willebrand Receptor in AML

The receptor for the von Willebrand Factor (CD42b) is one of the most major adhesive receptors expressed on the surface of circulating platelets, able to interact with several ligands, including the adhesive protein von Willebrand factor, the coagulation factors (thrombin, factors XI and XII), and the membrane glycoproteins (*p*-selectin and Mac-1) [46]. The reduced expression of CD42b in our patients might be interpreted as a particular reduction in the α chain of GpIb due to the downregulation during platelet activation [49,50,51]. Interestingly, Leinoe et al. [21] investigated if this decrease in CD42b in platelets under TRAP activation may occur because a downregulation of GpIb due to platelet activation in vivo. What we can say is that the same findings in vivo suggest that a broken activation mechanism could be involved in the expression of both *p*-selectin and granulophysin and the decreasing of GpIb, considered to be a regulatory mechanism, which may witness this effort to activate a defective platelet.

### 4.2. Fibrinogen Receptor

The resting form of the CD41/CD61 complex binds to immobilized fibrinogen, and, upon platelet activation, the complex becomes a receptor for soluble fibrinogen, fibronectin, the von Willebrand Factor, vitronectin and thrombospondin [52]. This receptor is also involved in platelet aggregation [53,54]. The anti CD41 antigen antibody reacts with GpIIb in the intact GpIIb-IIIa complex, blocking fibrinogen and platelet aggregation induced by thrombin, collagen and ADP. The reduction in the CD61 fluorescence level may be interpreted as a selective decrease in the β3 subunit of GpIIIa, which may be a cause of the aggregation defect. A defective β3 integrin phosphorylation, associated with altered outside-in signalling, was observed [55]. This may lead to an increase in the haemorrhagic syndrome of AML patients.

In AML, the low expression of GP1b on platelets could be explained by action of elastase released from myeloid blasts [56]. The interaction between platelets and AML blasts was proven by one study, and this interaction could produce functional changes and explain the presence of the dysfunctional platelet in AML patients [19]. A correlation between the low expression of CD62P, CD63 and the fibrinogen receptor and the high risk for haemorrhage in the next 7 days of follow up for AML patients was reported recently in platelets stimulated by TRAP. In AML patients defective signalling pathways were found that could explain the low activation of GPIIbIIIa, an abnormal conformation of this activated receptor that influences its function [3,11]. In other studies, a correlation was observed between the high expression of *p* selectin and GP IIbIIIa and haemorrhagic history [20]. In AML patients, the MPL expression on blasts was correlated with the severity of neutropenia and thrombocytopenia, especially in AML patients with t(8;21) genetic aberrancy [57].

### 4.3. Clinical and Laboratory Characteristics

The documented presence of the infection for the day before the study and/or the fever is associated with different types of bleeding, which can be clinically assessed as mild, significant or major. Recently, Vinholt et al. did not obtain any significant correlation between the infection and haemorrhagic risk of AML patients [3]. Gaydos et al. [58] found correlations between platelet numbers and all types of bleedings (mild, clinically significant and major) in acute leukemia patients. Lawrence et al. [59] obtained no correlation between bleeding and the number of platelets, or the minimal number of the platelets. Other studies, which focused on the same issue, analysed this in association with the clinical issue of prophylactic transfusion with platelet concentrate and stressed that the risk of an important haemorrhagic complication still exists [60,61]. The absence of any correlation between cutaneous haemorrhage and the number of platelets, in addition to the above-described aberrant expression of phenotypic surface platelets markers, emphasizes the role of functional and structural platelet defects in the haemorrhagic burst.

## 5. Conclusions

In our group of patients, we found a significant decrease in activation markers such as *p*-selectin and granulophysin, associated with the decrease in GpIb and β_3_ integrin, demonstrating that, in AML, there could be multiple defects of signalling transduction, leading to defects of adhesion, aggregation and the secretion of platelets, consequently leading to haemorrhagic syndrome independent of the number of platelets in the peripheral blood. These data are complementary to other already published results in demonstrating the multiple platelet defects of AML patients, suggesting that the risk of bleeding is directly correlated to the level of platelet defect.

In conclusion, the platelet behaviour in our study group offers important clues for postulating that flow cytometry analysis could be a very important method to identify those patients that are potential candidates to haemorrhagic disorders.

A limitation of our study is the small number of patients in the study group and the lack of genetic aberrancies data in AML patients, which could be of clinical interest in further research. Additionally, the study was not designed as a longitudinal study, and a follow up study could be of interest for more information regarding haemorrhagic risk.

## Figures and Tables

**Table 1:** Characteristics of patients with AML and controls.

	AML (N = 22)	C (N = 10)
Females/males	13/9	6/4
Age in years	48 (28–83)	24 (10–38)
Intake of NSAID	none	none
Intake of anticoagulants	1	none
Treatment with beta-lactam antibiotics	10 (45%)	none
Chemotherapy	2	none
Corticotherapy	10	none
Received haemostatic iv treatment ^b^	13	none
Received blood products a day before (erythrocyte concentrate/thrombocyte concentrate)	7 (2/5)	none
Platelet count × 10^9^/L ^a^	50 (2–782)	256 (143–400)
Haematocrit (%) ^a^	24 (17–38)	34 (32–42)
Leucocytes × 10^9^/L ^a^	3 (0.5–33.7)	5200 (4300–7000)
Peripheral blasts (%)	0–72%	none
Cutaneus haemorrhagic syndrome	13	none
Infection (localized or generalized)	11	none

NSAID, non-steroid anti-inflammatory drugs; ^a^ Data are presented as medians, with ranges in parentheses; ^b^ Haemostatic treatment: etamsylatum and carbazochromi salicylas.

**Table 2:** Platelets’ antigens distribution in AML patients and controls.

Markers/Cases	Median (25–75%)		*p* Value *
	C	AML	
% of CD62p positive	28.23 (18.7–30.12)	15.26 (7.51–26.88)	0.038
% of CD63 positive	40.78 (31.92–43.35)	14.11 (6.23–30.10)	0.001
% of CD42a positive	94.15 (78.2–97.8)	77.98 (60.14–94.92)	0.099
% of CD42b positive	79.45 (76.42–96.27)	72.96 (45.63–91.3)	0.047
% of CD61 positive	98.67 (98.16–98.88)	94.08 (74.45–96.78)	<0.01
% of CD41 positive	86.31 (73.53–94.21)	80.62 (55.51–92.14)	0.29

C, controls; AML, acute myeloid leukaemia. * *p* value represents the statistical significance of platelets expressing positive fluorescence analysis, in patients with acute leukaemia and control.

**Table 3:** Statistical correlations in AML group.

Correlation Pairs	Spearman’s Rho *	*p* Value
CD62p-CD63	0.695	0.000
CD63-Ht	0.598	0.003
CD 63-Blasts	0.447	0.037
CD42a-CD41	0.783	0.000
CD42b-CD42a	0.823	0.000
CD42b-CD61	0.570	0.007
CD42b-CD41	0.761	0.000
CD61-CD42a	0.758	0.000
CD61-CD42b	0.570	0.007
CD61-CD41	0.684	0.000
CD41-CD42a	0.783	0.000
CD41-CD42b	0.761	0.000
T^o^-Ht	0.559	0.007

*: Correlation is significant at the 0.05 level (2-tailed). T^o^ = temperature of patients (higher or normal). Ht = haematocrit.

**Table 4:** Platelets’ antigens distribution in AML patients with or without bleeding.

Markers	Cases and the Presence of Hemorrhage	Median (25–75%)	*p* Value
% of CD62p positive	AML NO AML YES	16.46 (7.38–47.32) 14.29 (0.86–65.18)	0.038
% of CD63 positive	AML NO AML YES	16.78 (5.12–45.76) 13.44 (0.69–56.15)	0.001
% of CD42a positive	AML NO AML YES	76.59 (1.44–95.81) 83.09 (35.49–98.28)	0.099
% of CD42b positive	AML NO AML YES	76.35 (12.94–95.31) 63.53 (34.85–95.05	0.047
% of CD61 positive	AML NO AML YES	87.51 (40.35–98.10) 94.25 (37.99–99.63)	<0.01
% of CD41 positive	AML NO AML YES	73.19 (21.42–93.06) 77.33 (27.34–97.27)	0.029

## Data Availability

All data are available, either analysed as figures and tables presented in the current manuscript; or as raw data upon request from any external collaborator or reviewer.
